# Direct epicardial evaluation of left atrial posterior wall isolation via an endocardial approach using a circular array pulsed field ablation catheter in a patient with atrial fibrillation

**DOI:** 10.1016/j.hrcr.2025.12.003

**Published:** 2025-12-10

**Authors:** Kazumasa Suga, Hiroyuki Kato, Taku Sakurai, Shinji Yamazoe, Kazuhito Tsuzuki, Hisashi Murakami

**Affiliations:** Department of Cardiology, Japan Community Healthcare Organization Chukyo Hospital, Nagoya, Aichi, Japan

**Keywords:** Atrial fibrillation, Direct epicardial mapping, Left atrial posterior wall, Pulsed field ablation, Residual epicardial conduction, Transmural lesion

**Key Teaching Points**

• During left atrial posterior wall isolation (LAPWI), a circular array pulsed field ablation (PFA) catheter can create transmural lesions across most of the posterior wall.
• Achieving complete transmurality with the circular array PFA catheter can be challenging in certain regions, potentially allowing persistent epicardial conduction.
• Further investigation of technical parameters, such as optimal application interval, catheter orientation, and the effect of multiple applications, is warranted to establish a strategy for consistently achieving transmural LAPWI with the circular array PFA catheter.

## Introduction

Pulmonary vein isolation (PVI) is the cornerstone of catheter ablation for atrial fibrillation (AF).^1^ However, the benefit of adjunctive left atrial posterior wall isolation (LAPWI) in persistent AF remains controversial. Randomized trials have not shown consistent benefit of adding LAPWI to PVI.^2^ Guidelines advise against routine LAPWI, reserving it for selected cases.^3^ In contrast, subgroup data suggest that LAPWI may reduce recurrence in patients with high-frequency activity on the left atrial posterior wall (LAPW).^4^

Recently, pulsed field ablation (PFA) has emerged as a nonthermal, tissue-selective modality that potentially reduces injury to adjacent structures such as the esophagus.^5^ Favorable 1-year outcomes are reported after PVI and LAPWI using a pentaspline PFA catheter.^6^

Nontransmural lesions on the LAPW can preserve endo-epicardial asynchronous activity, potentially sustaining AF drivers.^7^ Achieving transmural lesions may be necessary for durable ablation. However, clinical evidence for transmural LAPW lesions by PFA is limited, and comparable circular array PFA catheter evaluations are lacking, despite epicardial assessments with the pentaspline catheter.^8^

Herein, LAPWI was performed using a circular array PFA catheter under simultaneous direct epicardial electrogram monitoring to assess LAPW transmurality.

## Case report

A 71-year-old man presented with decompensated heart failure (New York Heart Association functional class III) due to symptomatic persistent AF lasting 4 months. He had heart failure, hypertension, and diabetes mellitus and had undergone cardiac resynchronization therapy–defibrillator implantation 4 years earlier for complete atrioventricular block and a left ventricular ejection fraction of 28%. At that time, coronary angiography revealed no significant coronary stenosis, indicating a nonischemic etiology of his left ventricular dysfunction.

On admission, laboratory testing showed a creatinine level of 0.9 mg/dL and a B-type natriuretic peptide level of 515 pg/mL. Echocardiography showed a left ventricular ejection fraction of 52%, a left atrial diameter of 43 mm, and pericardial effusion with early diastolic right ventricular collapse. The patient was treated with diuretics and electrical cardioversion, which terminated AF. However, AF recurred shortly thereafter while his heart failure symptoms persisted and the pericardial effusion remained unchanged. Given symptomatic persistent AF, catheter ablation was considered reasonable (Class IIa recommendation) to improve symptoms by restoring atrioventricular synchrony.^1^^,^^3^ Therefore, simultaneous catheter ablation for AF and pericardial drainage were scheduled. Written informed consent was obtained from the patient for the publication of this case report, including the use of anonymized clinical data and any associated images or videos.

AF ablation was performed using a circular array PFA catheter (PulseSelect, Medtronic, Minneapolis, MN). 3-dimensional electroanatomic mapping was conducted with the EnSite X system (Abbott, Abbott Park, IL) using the Advisor HD Grid Mapping Catheter, Sensor Enabled (Abbott; 3-mm electrode spacing). The Omnipolar Technology Near-Field algorithm was used for electrogram recording, and a peak frequency map was obtained. During the procedure, the cardiac resynchronization therapy–defibrillator was programmed to VVI mode at 40 beats/min.

**Figure 1:**
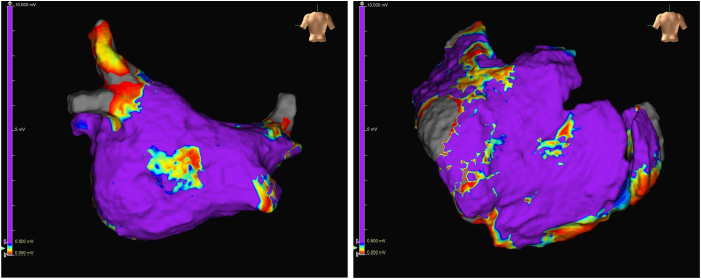
Endocardial and epicardial voltage maps before pulsed field ablation. Endocardial (left) and epicardial (right) voltage maps of the left atrial posterior wall. Voltage scale: *purple*, ≥0.5 mV; *gray*, <0.05 mV.

Under general anesthesia, coronary angiography was performed for ischemic reevaluation in decompensated heart failure and showed no significant stenosis. Subsequently, pericardiocentesis was conducted via a subxiphoid approach, and drainage of 490 mL of serous pericardial fluid resulted in a 15-mm Hg increase in systolic blood pressure. An 8.5-F flexible sheath was inserted into the pericardial cavity for epicardial mapping. Before PFA, endocardial and epicardial LAPW mapping was performed using an HD Grid catheter (Figure 1). Electroanatomic mapping revealed minimal low-voltage areas but confirmed rapid LAPW activity (shortest cycle length <140 ms) during spontaneously initiated AF.^4^ Given this finding and his decompensated heart failure, preventing recurrence was clinically imperative. Considering the Class IIb recommendation^3^ and limited evidence, we performed adjunctive LAPWI for maximal durable success.

PVI was performed sequentially in the left superior pulmonary vein, followed by the left inferior pulmonary vein, right superior pulmonary vein, and right inferior pulmonary vein. At each pulmonary vein ostium and antrum, the catheter was rotated 90° between applications, and 4 applications were delivered per site, with additional applications to the carina. The total number of applications was 40 (13 for the left superior pulmonary vein, 7 for the left inferior pulmonary vein, 10 for the right superior pulmonary vein, and 10 for the right inferior pulmonary vein).

**Figure 2:**
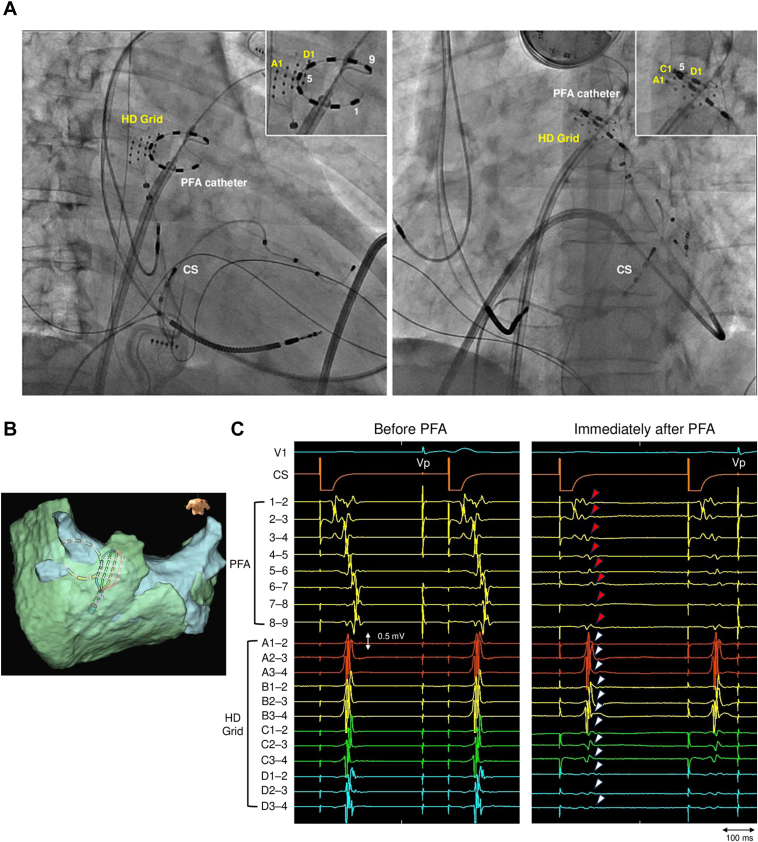
Catheter positioning and electrogram changes with pulsed field ablation (PFA) along the left atrial posterior wall roof. **A:** Fluoroscopic images in the right anterior oblique (left) and left anterior oblique (right) views. The positional relationship between the epicardial HD Grid catheter and the endocardial PFA catheter is shown. *Yellow text* in the upper right panel indicates HD Grid electrode numbers; *white text* indicates PFA catheter electrode numbers. **B:** 3-dimensional map indicating the relative positions of the endocardial PFA catheter and epicardial HD Grid catheter at the application site. **C:** Endocardial and epicardial electrograms before (left) and immediately after (right) a single PFA application during coronary sinus (CS) pacing (cycle length 400 ms). On the endocardium, the PFA catheter electrograms (*red arrows*) showed elimination of high-frequency components after application. In contrast, on the epicardium, the HD Grid electrograms (*white arrows*) showed a distance-dependent effect from the nearest PFA site (electrodes D): electrograms were abolished at electrodes D, showed elimination of high-frequency components at electrodes C (3 mm), were slightly attenuated at electrodes B (6 mm), and remained unchanged at electrodes A (9 mm). Vp indicates pacing from the cardiac resynchronization therapy defibrillator.

After PVI, linear PFA targeted the LAPW roof and floor. The application interval was set to achieve 50% overlap between the adjacent circular ablation footprints, with 1 application delivered at each site. Catheter contact was assessed under fluoroscopic guidance. A single application was delivered when electrode 5 of the PFA catheter on the endocardial side was positioned close to electrodes D of the HD Grid catheter on the opposite epicardial side (Figure 2A and 2B). Immediately after application, high-frequency components disappeared in the electrograms recorded by the PFA catheter (Figure 2C). On the epicardial side, the electrograms of the nearest electrodes D were eliminated while the high-frequency components disappeared at electrodes C (3 mm away from electrodes D). In contrast, at electrodes B (6 mm away from electrodes D), only a slight decrease in the amplitude of the high-frequency components was observed while no electrogram change was observed at electrodes A (9 mm away from electrodes D) (Figure 2C). In total, 12 applications were performed for the roof and 6 for the floor.

**Figure 3:**
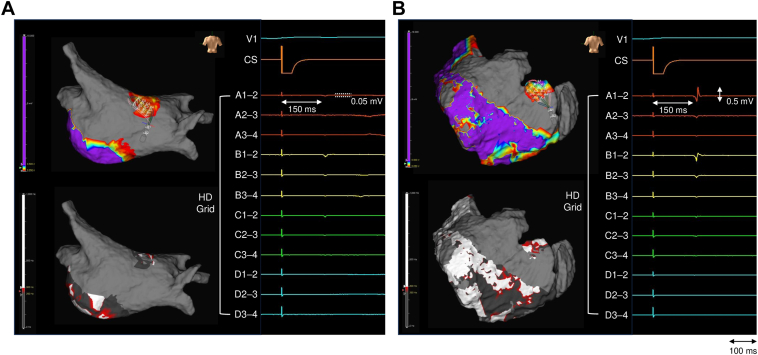
Voltage and peak frequency maps after the first pass along the left atrial posterior wall roof and floor, with representative electrograms. **A:** Endocardial side: top, voltage map; bottom, peak frequency map. The right panel displays representative HD Grid electrograms. **B:** Epicardial side: top, voltage map; bottom, peak frequency map. Representative HD Grid electrograms are shown (right). Voltage scale: *purple*, ≥0.5 mV; *gray*, <0.05 mV. Peak frequency map scale: *white*, ≥300 Hz; *dark gray*, <250 Hz. All maps were obtained during coronary sinus pacing.

**Figure 4:**
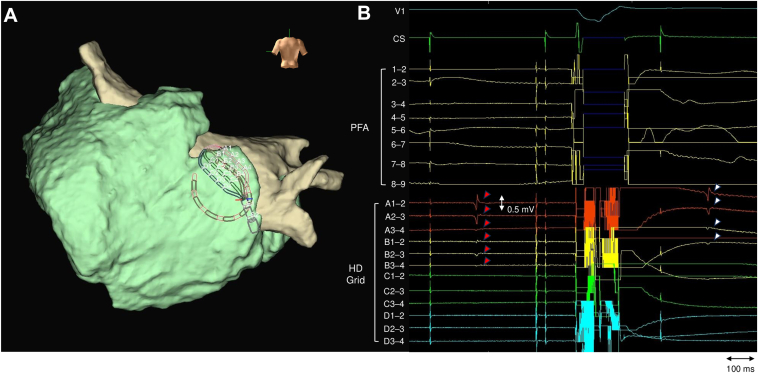
Additional pulsed field ablation (PFA) at the right roof: 3-dimensional map and epicardial electrograms before and after the application. **A:** 3-dimensional map showing the relative positions of the endocardial PFA catheter and opposing epicardial HD Grid catheter on the right roof segment. **B:** Epicardial HD Grid electrograms recorded before (*red arrows*) and immediately after (*white arrows*) the additional PFA application. Maps were obtained during coronary sinus pacing.

**Figure 5:**
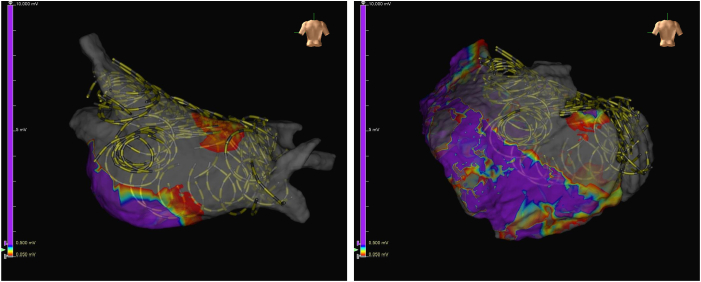
Cumulative map of pulsed field ablation catheter positions after pulmonary vein isolation and left atrial posterior wall isolation. Voltage map displaying all application sites. Voltage scale: *purple*, ≥0.5 mV; *gray*, <0.05 mV.

After PFA for the roof and floor, electroanatomic mapping with the HD Grid catheter revealed residual electrograms on the endocardial aspect of the right roof with amplitudes of 0.05 mV (Figure 3A and Supplemental Video 1). Residual electrograms in the same phase were also observed on the opposite epicardial aspect, with amplitudes higher than those on the endocardium (Figure 3B and Supplemental Video 2). On the peak frequency map with the Omnipolar Technology Near-Field algorithm (a lower threshold of 250 Hz), the peak frequency values on the epicardial side were higher than those on the endocardial side. These residual electrograms were not detectable when the PFA catheter was positioned on the endocardial side. Despite 4 additional PFA applications to the residual conduction gap in the right roof, epicardial electrograms remained unchanged (Figure 4). Residual electrograms persisted on the epicardial aspect of the upper right LAPW. A total of 22 PFA applications were delivered for LAPWI (Figure 5). Epicardial ablation was not performed because of the procedural invasiveness.

Hereditary transthyretin cardiac amyloidosis (ATTRv-CA) was confirmed by myocardial biopsy and genetic testing after serum and urine assays excluded light chain amyloidosis. No periprocedural complications occurred. At 3 months, no AF recurrence or heart failure hospitalization occurred. Although a moderate amount of pericardial effusion reaccumulated, it did not progress to cardiac tamponade, and his heart failure symptoms improved to New York Heart Association functional class I. As initiation of subcutaneous vutrisiran was planned for his ATTRv-CA, the effusion was managed with continued monitoring.

## Discussion

This case report suggests that incomplete transmural lesions with persistent epicardial conduction may occur during endocardial LAPWI using a circular array PFA catheter. Epicardial assessment was opportunistically performed during therapeutic pericardial drainage, not as a routine strategy. While endocardial assessment suggested transmural lesions in most areas, epicardial evaluation revealed persistent residual electrograms at the upper right LAPW. This finding may have clinical relevance because failure to consistently achieve transmural lesion formation owing to anatomical and technical challenges may partly explain the variability among reports regarding the heterogeneous outcomes of adjunctive LAPWI strategies.

A single circular array PFA application from the endocardial side produced a distance-dependent reduction in epicardial electrograms obtained from the HD Grid catheter (Figure 2C). The electrograms closest to electrodes D were abolished. At electrodes C (3 mm from electrodes D), the high-frequency components disappeared, whereas residual electrograms persisted at electrodes B and A (6 and 9 mm from electrodes D, respectively). An ex vivo study using a prototype circular array catheter showed a mean lesion width of 9.4 mm—approximately 4.7 mm extending from the contact point on each side—and a mean depth of 4.3 mm.^9^ These benchmarks are consistent with our observations and may serve as a practical reference for determining application intervals during linear ablation.

On the epicardial side, the residual electrograms exhibited greater amplitudes and higher peak frequency values compared with those on the endocardial side (Figure 3). As high peak frequency values indicate a near-field origin directly beneath the recording electrode,^10^ the residual electrograms observed were likely of epicardial origin. Furthermore, the residual electrograms were not detected by the PFA catheter but were identified with the HD Grid catheter (0.05-mV threshold), limiting confirmation with the PFA catheter alone.

Several factors may account for the residual electrograms confined to the epicardial aspect. First, regarding LAPW anatomy, the septopulmonary bundle traverses the left atrial roof, and the roof wall thickness ranges from 3.5 to 6.5 mm (mean 4.5 ± 0.6 mm).^11^, ^12^, ^13^ In the right-roof region, epicardial conduction persisted despite repeated PFA applications (Figure 4), likely attributable to these anatomical factors. This underscores the limitation of the circular array PFA catheter in achieving transmural lesion formation at specific anatomical sites. Second, catheter design may contribute. The pentaspline catheter, when deployed in its flower configuration, is designed to achieve planar and uniform electrode-tissue contact against the LAPW. The study protocol, which specified a 50% overlap of application sites, aimed to create contiguous lesions.^6^ In contrast, the presence of the nose of the circular array catheter at the distal tip can hinder uniform contact of the entire electrode array with the tissue. Consequently, lesions may form as discrete linear lines around individual electrodes. Therefore, achieving contiguous lesions comparable with those from the pentaspline catheter may require fine adjustments to the orientation of the circular array catheter or a higher number of applications. Third, electrode-tissue contact may have contributed. Because even small increases in distance can markedly reduce PFA lesion depth,^9^ suboptimal contact during some applications cannot be excluded.

A few limitations should be noted. Confirmed ATTRv-CA and pericardial effusion might have introduced anatomical and physiological differences compared with typical AF cohorts. Exit block assessment with high-output pacing was not performed, preventing full evaluation of the functional significance of residual epicardial conduction. Intracardiac echocardiography was not used to assess catheter contact. Distance-dependent attenuation was assessed at only 1 posterior wall segment (Figure 2C). Moreover, whether focal residual epicardial electrograms after LAPWI are arrhythmogenic remains unclear. The long-term durability of LAPWI created with a circular array PFA catheter could not be determined. Future studies are warranted to clarify the clinical significance of complete transmurality in LAPWI and the clinical relevance of residual epicardial conduction.

## Conclusion

Direct epicardial evaluation during LAPWI using a circular array PFA catheter highlighted the potential for residual epicardial conduction, likely attributable to anatomical and technical factors. Real-time epicardial assessment of electrogram reduction during endocardial LAPWI using a circular array PFA catheter is feasible.

## Disclosures

The authors have no conflicts of interest to disclose.

## Appendix Supplementary Data

Supplementaly Material

## Footnotes

Appendix Supplementary data associated with this article can be found in the online version at https://doi.org/10.1016/j.hrcr.2025.12.003.
