# Supplementary material for: Direct epicardial evaluation of left atrial posterior wall isolation via an endocardial approach using a circular array pulsed field ablation catheter in a patient with atrial fibrillation
Source: HeartRhythm Case Rep. 2025 Dec 10;12(3):312–7. doi: 10.1016/j.hrcr.2025.12.003 (PMC13005083; doi:10.1016/j.hrcr.2025.12.003)
Supplement: Supplementaly Material [file mmc1.docx]

**Supplemental videos. Sparkle maps on voltage maps after first-pass PFA of the LAPW roof and floor**

Supplemental Video 1. Endocardial sparkle map

Supplemental Video 2. Epicardial sparkle map

Voltage scale: purple ≥0.5 mV; gray <0.05 mV.

LAPW, left atrial posterior wall; PFA, pulsed field ablation.
